# Comparison of Cardiorespiratory Fitness Prediction Equations and Generation of New Predictive Model for Patients with Obesity

**DOI:** 10.1249/MSS.0000000000003463

**Published:** 2024-05-15

**Authors:** MARCO VECCHIATO, ANDREA AGHI, RAFFAELE NERINI, NICOLA BORASIO, ANDREA GASPERETTI, GIULIA QUINTO, FRANCESCA BATTISTA, SILVIA BETTINI, ANGELO DI VINCENZO, ANDREA ERMOLAO, LUCA BUSETTO, DANIEL NEUNHAEUSERER

**Affiliations:** 1 Sports and Exercise Medicine Division, Department of Medicine, University of Padova, Padova, ITALY; 2 Fisioterapia Osteopatia Raimondi di Giovanni e Daniele, Selvazzano Dentro, Padova, ITALY; 3 Center for the Study and Integrated Treatment of Obesity (CeSTIO), Internal Medicine 3, Department of Medicine, University Hospital of Padova, Padova, ITALY

**Keywords:** CRF, V̇O_2_, OBESE, TREADMILL, MODEL, CARDIOPULMONARY EXERCISE TESTING

## Abstract

**Purpose:**

Cardiorespiratory fitness (CRF) is a critical marker of overall health and a key predictor of morbidity and mortality, but the existing prediction equations for CRF are primarily derived from general populations and may not be suitable for patients with obesity.

**Methods:**

Predicted CRF from different non-exercise prediction equations was compared with measured CRF of patients with obesity who underwent maximal cardiopulmonary exercise testing (CPET). Multiple linear regression was used to develop a population-specific nonexercise CRF prediction model for treadmill exercise including age, sex, weight, height, and physical activity level as determinants.

**Results:**

Six hundred sixty patients underwent CPET during the study period. Within the entire cohort, *R*^2^ values had a range of 0.24 to 0.46. Predicted CRF was statistically different from measured CRF for 19 of the 21 included equations. Only 50% of patients were correctly classified into the measured CRF categories according to predicted CRF. A multiple model for CRF prediction (mL·min^−1^) was generated (*R*^2^ = 0.78) and validated using two cross-validation methods.

**Conclusions:**

Most used equations provide inaccurate estimates of CRF in patients with obesity, particularly in cases of severe obesity and low CRF. Therefore, a new prediction equation was developed and validated specifically for patients with obesity, offering a more precise tool for clinical CPET interpretation and risk stratification in this population.

Cardiorespiratory fitness (CRF) indicates the subjects’ functional capacity and is a marker of general health associated with morbidity and mortality risk (1,2). Therefore, recent scientific statements suggest that CRF should be routinely evaluated in clinical settings (3). The gold standard to assess CRF is directly measuring maximal oxygen consumption (V̇O_2max_) through cardiopulmonary exercise testing (CPET) (4). However, because of the limited availability of performing CPET, CRF is frequently also estimated with nonexercise equations.

In order to use these predicted CRF values in a clinical setting, it is essential to evaluate and validate the underlying equations. Indeed, by creating precise reference values for specific populations, a better interpretation of CPET data can be obtained. A recent work has compared the different CRF prediction equations in apparently healthy subjects (5), but no study has yet specifically investigated this issue in populations with chronic diseases.

Obesity is a pathological condition whose incidence has reached pandemic proportions (6,7), and it is one of the main risk factors for cardiovascular diseases, diabetes, musculoskeletal disorders, and some types of cancer (8). Accordingly, patients with obesity should aim for a good CRF to reduce morbidity and mortality risk. Thus, for an adequate prognostic risk stratification in clinical settings, the interpretation of CPET data requires reference values that define the normal CRF range specifically for patients with obesity, because the current reference standards are based on a general population (9). However, the few studies conducted on patients with obesity had a limited sample size (10) or have not been adopted in clinical practice (11). Consequently, current CPET reference standards are not intended for patients with obesity, and their application in clinical practice can lead to significant interpretation difficulties, errors, or limitations.

Thus, the aim of the study was to evaluate the accuracy of the present nonexercise CRF prediction equations by comparing their outputs with measured CRF values in a large cohort of patients with obesity. Finally, the second aim of the study was to generate and validate a novel model for CRF predictions for this specific population.

## METHODS

### Study participants

The present study is an analysis on data from CPET performed from November 2017 to April 2022 in patients with obesity at the Sports and Exercise Medicine Division of the University of Padova. All patients underwent a functional evaluation including CPET as part of the regional diagnostic–therapeutic pathway of clinical assistance for obesity, defined as patients having a body mass index (BMI) >30 kg·m^−2^ (7). Patients were considered belonging to class I obesity for BMI values between 30.0 and 34.9 kg·m^−2^, class II for BMI values between 35.0 and 39.9 kg·m^−2^, and class III for BMI values equal to or greater than 40.0 kg·m^−2^. All data including the results of CPET were collected and managed in a research database, the “Cardiopulmonary Exercise test registry for Patients with Obesity”. This study was performed in accordance with the Declaration of Helsinki and approved by the “Padova Ethical Committee for Clinical Research” (99n/AO/21); all participants provided written informed consent.

### Predicted CRF: V̇O_2max_ predicting equations

The equations for estimating predicted CRF were obtained through a literature search conducted using the MEDLINE electronic database with the following terms: “predicted maximal oxygen uptake,” “predicted maximal oxygen consumption,” “estimated maximal oxygen uptake,” “estimated maximal oxygen consumption,” “predicted V̇O_2max_,” “predicted V̇O_2peak_,” “estimated V̇O_2max_,” “estimated V̇O_2peak_,” “non-exercise testing,” and “non-exercise prediction” (to June 15, 2023). The inclusion criteria for the equations in this study were as follows:

- equations estimating CRF in both genders;
- treadmill as the exercise modality used to create the equation;
- prediction equations included at least weight, BMI, or waist circumference (WC) as variables to determine CRF;
- variables within the equation were available from the data collected in our center (e.g., excluded equations requiring percentage fat or lean mass).

Additional equations were included from previous reviews and respective references (Supplemental Table 1, Supplemental Digital Content 1, Nonexercise prediction equations to estimate CRF, http://links.lww.com/MSS/D19).

Patients with acute or chronic heart, vascular, or lung diseases were excluded from the study. Comorbidities frequently associated with obesity such as diabetes, arterial hypertension, and/or dyslipidemia were not considered as exclusion criteria. The variables of each equation were matched to our cohort database with appropriate adjustments for physical activity level and smoking history. The equations estimating maximal exercise capacity through METs were converted to V̇O_2max_ (mL·kg^−1^·min^−1^) through multiplying by 3.5.

Variables within the equations included sex, age, height, weight, BMI, WC, smoking history, resting heart rate (HR), dyslipidemia, arterial hypertension, diabetes, and the physical activity level. The latter was registered as total weekly hours according to World Health Organization (WHO) recommendations—inactive: no regular physical activity; low active: <150 min·wk^−1^ of moderate-vigorous intensity physical activity; active: between 150 and 300 min·wk^−1^ of moderate-vigorous intensity physical activity; very active: >300 min·wk^−1^ of moderate-vigorous intensity physical activity (12). Smoking status/history was recorded as a dichotomous variable (yes or no) and as the number of cigarettes smoked daily. The conversions between the various physical activity scales and the smoking scales are shown in Supplemental Table 2 (Supplemental Digital Content 1, Table converting physical activity level and smoking history between equations, http://links.lww.com/MSS/D19). No CRF was estimated when there was a lack of data regarding a parameter of an equation.

### Measured CRF: V̇O_2max_ direct measurement

Participants performed an incremental, maximal electrocardiogram-monitored CPET (Masterscreen-CPX; Vyaire, Yorba Linda, CA) on a treadmill (T170 DE; h/p Cosmos, Nussdorf-Traunstein, Germany) using a standardized protocol (modified Bruce ramp protocol) (13). Torso-lead electrocardiogram was recorded at rest, during the exercise phase, and during recovery (Cardioline US, San Diego, CA). HR was recorded both at rest and at peak exercise, providing it also as percentages of maximum HR. Continuous monitoring of the electrocardiogram was performed throughout the test, and the respiratory gas exchange and ventilation were monitored breath by breath during the whole test (data averaged for every 20 s). For this study, CRF was defined as V̇O_2max_, i.e., the highest value of V̇O_2_ attained in a 30-s interval before peak exercise. Criteria of exhaustion were a Borg rating of perceived exertion ≥18/20 associated with a respiratory exchange ratio (RER) >1.1. All tests were conducted under the supervision of a specialized physician in sports and exercise medicine. Tests were excluded if participants did not achieve a RER of at least 1.1 or were taking beta-blockers.

### Statistical analyses

Normally distributed data were summarized as mean ± standard deviation, non-normally distributed data as median and interquartile range, and binary/categorical data as percentages, as appropriate. An analysis of variance test was performed for comparison between the different obesity classes. Chi-squared tests were used to analyze categorical data. Multiple comparisons between measured and predicted CRF were examined using the Benjamini–Hochberg procedure: *P* values from *t*-tests of the measured and predicted CRF were ranked and compared with a critical value with a false discovery rate of 5% (14). The relationship between the different prediction equations and measured CRF was examined by calculating the coefficient of determination (*R*^2^) and the standard error of estimates (SEE). Bland–Altman plots were created to visualize the relationship between the different prediction equations and directly measured CRF. Participants were classified as having “low” CRF if they were below the 33rd percentile, “intermediate” CRF if between the 33rd and 66th percentile, and “high” CRF if above the 66th percentile. Additional analyses also examined participants in age ranges (younger: <40 yr, middle aged: 40 to 50 yr, older: >50 yr).

Multiple linear regression was used to develop a nonexercise prediction model for treadmill exercise. The dependent variable was CRF expressed as milliliters per minute. The independent variables were sex (0: female, 1: male), age (yr), weight (kg), height (cm), and physical activity level (0: inactive; 1: active, considered from low to high physical activity level). The 10-fold and Monte Carlo cross-validation methods were applied with caret package of R-Studio to evaluate the out-of-sample prediction errors for the models. Congruence of the prediction models was examined by comparing the model fit statistics. Statistical significance was set at *P* < 0.05. All analyses were performed using R-Studio, Version 2023.03.1+446 and SAS.

## RESULTS

**FIGURE 1:**
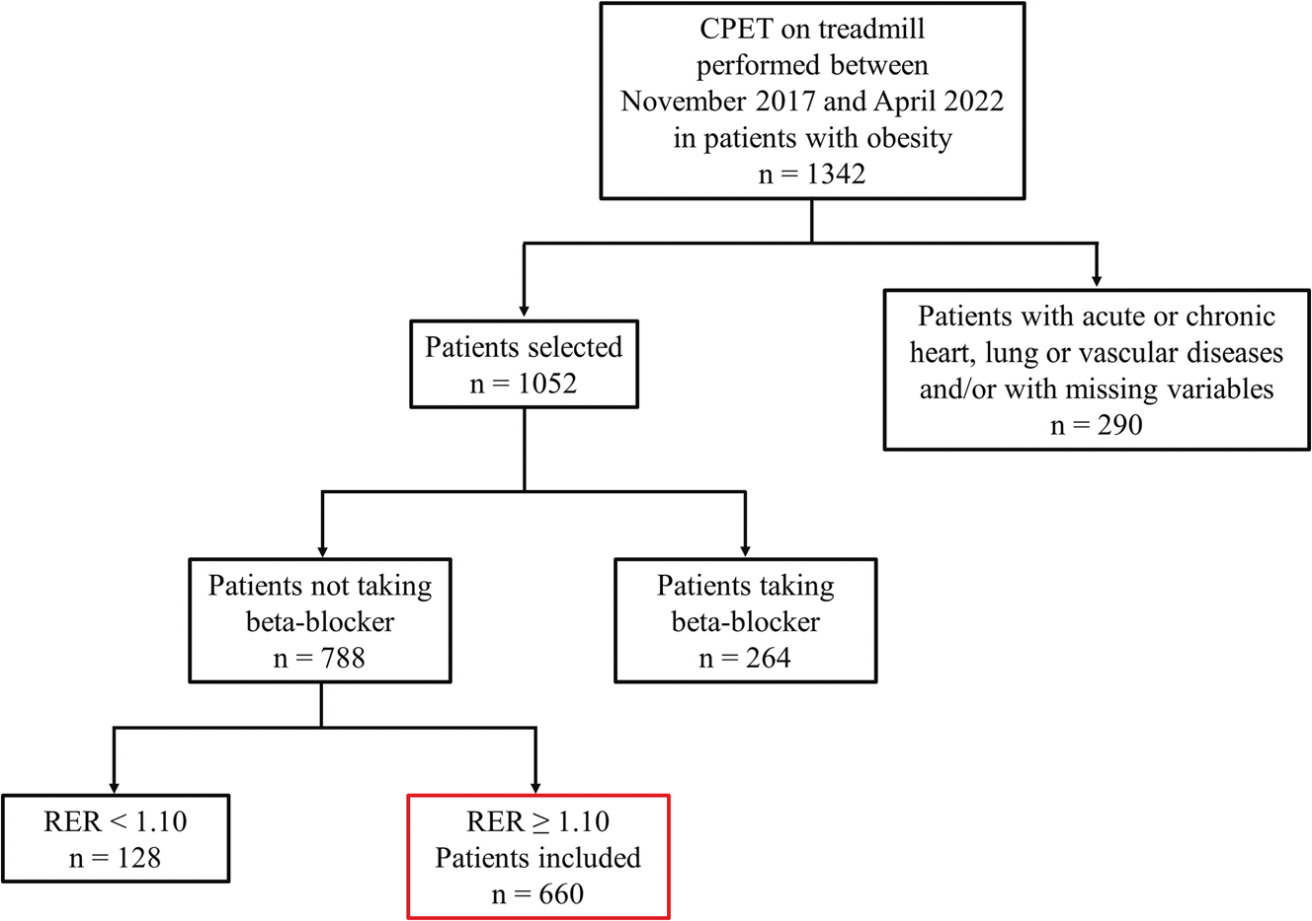
Flow chart of the included patients.

From a total of 1342 patients who underwent CPET during the study period, a cohort of 660 patients was selected (Fig. 1). Anthropometric and CPET characteristics of the study sample, grouped by obesity classes, are shown in Table 1.

**TABLE 1:** Descriptive characteristics of included patients.

	All (N = 660)	Class I Obesity (N = 120)	Class II Obesity (N = 157)	Class III Obesity (N = 383)
**Sex (male%)**	204 (30.9%)	45 (37.5%)	39 (24.8%)	120 (31.3%)
**Age (yr)**	44.2 ± 11.3	46.8 ± 12.2	46.3 ± 9.77	42.4 ± 11.3
**Height (cm)**	166.6 ± 9.5	167.9 ± 9.51	165.5 ± 9.40	166.7 ± 9.62
**Weight (kg)**	116.6 ± 23.9	92.3 ± 11.3	104 ± 11.7	130 ± 21.6
**WC (cm)**	126.6 ± 16.6	108.9 ± 10.9	118.1 ± 11.9	133.5 ± 14.9
**BMI (kg·m^−2^)**	41.8 ± 6.8	32.6 ± 1.5	37.7 ± 1.4	46.4 ± 4.9
**Physical activity level**				
No regular physical activity	396 (60%)	55 (45.8%)	95 (60.5%)	246 (64.2%)
150 min·wk^−1^	175 (26.5%)	41 (34.2%)	37 (23.6%)	97 (25.3%)
150 to 300 min·wk^−1^	48 (7.3%)	17 (14.2%)	14 (8.9%)	17 (4.4%)
>300 min·wk^−1^	41 (6.1%)	17 (5.8%)	11 (7.0%)	23 (6.0%)
**Smoker (%)**	88 (13.3%)	17 (14.2%)	16 (10.2%)	55 (14.4%)
**Arterial hypertension (%)**	213 (32.3%)	30 (25.0%)	51 (32.5%)	132 (34.5%)
**Diabetes (%)**	133 (20.2%)	22 (18.3%)	31 (19.8%)	80 (20.9%)
**Dyslipidemia (%)**	133 (20.2%)	21 (17.5%)	34 (21.7%)	78 (20.3%)
**Arthropathies (%)**	125 (18.9%)	9 (7.5%)	31 (19.8%)	85 (22.2%)
**HR rest (bpm)**	77.8 ± 12.6	68.8 ± 11.0	76.8 ± 11.6	80.9 ± 12.0
**HR max (bpm)**	162 ± 17.5	160 ± 22.8	163 ± 15.4	162 ± 16.3
**RER peak**	1.21 ± 0.09	1.25 ± 0.12	1.21 ± 0.07	1.20 ± 0.07
**V̇O_2peak_ (mL·kg^−1^·min^−1^)**	20.2 ± 3.87	23.2 ± 4.59	21.3 ± 3.55	18.8 ± 2.97
**V̇O_2peak_ (mL·min^−1^)**	2330 ± 536	2150 ± 527	2220 ± 488	2440 ± 534

A total of 21 nonexercise prediction equations were considered, and their formulae were reported in Supplemental Table 1 (Supplemental Digital Content 1, http://links.lww.com/MSS/D19). Supplemental Tables 3 to 6 (Supplemental Digital Content 1, Predicted CRF vs measured CRF for all patients with obesity according to gender, to obesity classes, to age groups, and to CRF groups, http://links.lww.com/MSS/D19) provide details of the population and prediction equation results according to sex, obesity classes, age, and CRF percentile groups, respectively. All predicted CRF values correlated with the measured CRF (*P* < 0.001) for the entire cohort as well as for different sex, obesity classes, age, and CRF groups. Within the entire cohort, the *R*^2^ values had a range of 0.24 to 0.46 with SEE ranging from 2.84 to 3.26 mL·kg^−1^·min^−1^. The range of *R*^2^ and SEE values was 0.33 to 0.44 and 3.11 to 3.51 mL·kg^−1^·min^−1^ for males and 0.28 to 0.40 and 2.65 to 2.91 mL·kg^−1^·min^−1^ for females, respectively. Statistically significant difference between predicted and measured CRF was evident for 19 equations when examining the entire cohort, 14 equations when examining only males, and 18 equations when examining only females. Within each obesity class, the range values of *R*^2^ were 0.16 to 0.45, 0.14 to 0.31, and 0.13 to 0.34 for class I, class II, and class III, respectively. Within each age group, the range values of *R*^2^ were 0.30 to 0.54, 0.22 to 0.44, and 0.16 to 0.46 for the younger, middle-aged, and older groups, respectively. Within each CRF group, the range values of *R*^2^ were 0.03 to 0.12, 0.01 to 0.06, and 0.15 to 0.38 for the lower, intermediate, and higher CRF groups, respectively. Supplemental Figure 1 (Supplemental Digital Content 2, http://links.lww.com/MSS/D20) shows the Bland–Altman plots for each equation included.

**FIGURE 2:**
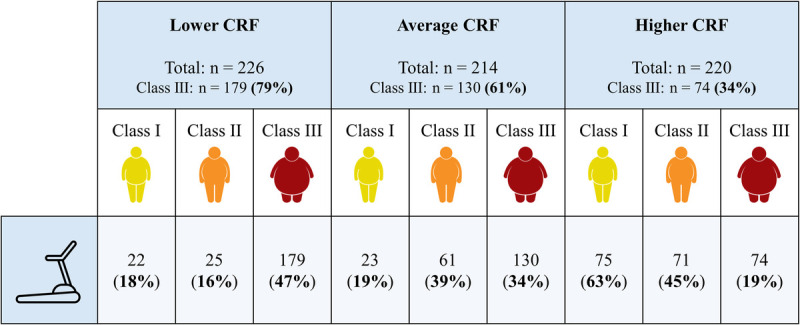
Obesity classes compared with measured CRF values. The percentage of class III obesity patients decreases as the CRF increases (79% with low CRF vs 34% in high CRF). Similarly, among class III obesity, almost half are classified as being in the lower CRF (47%).

The relationship between measured CRF and obesity classes in our cohort is shown in Figure 2. Patients belonging to the lowest CRF tertile are more frequently in class III than those belonging to the remaining tertiles. Table 2 presents the percentage of participants correctly assigned to the measured CRF tertiles when using the predicted CRF. The selected prediction equations correctly categorized only 50% of patients (43% of class III). Consequently, the prediction equations incorrectly placed 28% of participants into a higher CRF group (33% of class III) and 22% into a lower CRF group (24% of class III).

**TABLE 2:** Percentage of patients classified according to measured and predicted CRF.

	Low Measured CRF			Average Measured CRF			High Measured CRF		
	Low predicted CRF	Average predicted CRF	High predicted CRF	Low predicted CRF	Average predicted CRF	High predicted CRF	Low predicted CRF	Average predicted CRF	High predicted CRF
Jackson et al., 1990 (15)	**92**	4	4	81	**7**	12	42	15	**43**
Wasserman et al., 1994 (16)	**45**	26	29	22	**42**	36	4	24	**72**
Whaley et al., 1995 (17)	**54**	17	28	22	**25**	53	7	9	**84**
Matthews et al., 1999 (18)	**65**	18	17	36	**25**	39	11	12	**77**
Jurca et al., 2005 (ACLS) (19)	**73**	14	13	49	**20**	31	19	14	**67**
Jurca et al., 2005 (ADNFS) (19)	**51**	16	33	21	**18**	61	4	9	**87**
Jurca et al., 2005 (NASA) (19)	**77**	12	11	57	**16**	27	21	14	**65**
Wier et al., 2006 (BMI) (20)	**83**	10	7	63	**18**	19	26	15	**58**
Wier et al., 2006 (WC) (20)	**76**	9	15	58	**15**	27	27	10	**63**
Nes et al., 2011 (21)	**28**	16	55	9	**14**	77	3	7	**90**
Cáceres et al., 2012 (22)	**54**	20	26	30	**22**	48	11	14	**75**
Jackson et al., 2012 (BMI with five levels) (23)	**44**	18	38	21	**14**	64	8	9	**83**
Jackson et al., 2012 (BMI with two levels) (23)	**44**	18	38	19	**17**	64	7	10	**83**
Jang et al., 2012 (model 1) (24)	**0**	0	100	0	**0**	100	0	0	**100**
Jang et al., 2012 (model 2) (24)	**0**	0	100	0	**0**	100	0	0	**100**
Baynard et al., 2016 (BMI) (25)	**79**	9	12	57	**18**	25	22	17	**61**
Baynard et al., 2016 (WC) (25)	**79**	6	15	64	**14**	22	32	14	**54**
Myers et al., 2017 (26)	**67**	11	22	38	**20**	42	11	14	**75**
De Souza et al., 2018 (27)	**83**	10	7	66	**17**	17	30	17	**53**
Nevill et al., 2018 (additive linear model) (28)	**75**	10	15	47	**27**	26	19	16	**65**
Nevill et al., 2018 (allometric model) (28)	**58**	25	17	30	**39**	31	9	21	**70**

Patients were divided into tertiles based on the measured CRF. Correct classifications are displayed in bold.

ACLS, equation based on data from the Aerobics Center Longitudinal Study; ADNFS, Allied Dunbar National Fitness Survey; NASA, National Aeronautics and Space Administration.

**FIGURE 3:**
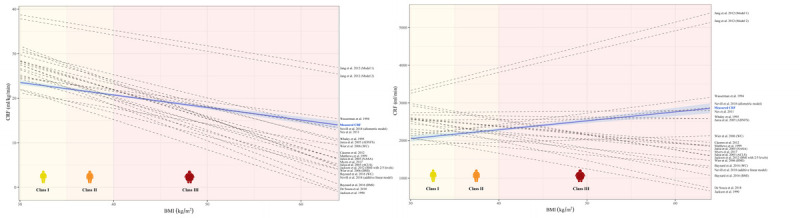
Predicted CRF of the included equations versus measured CRF across the obesity classes. The graphs represent the relationship between BMI and relative (A; on the left) and absolute CRF (B; on the right). The *blue line* represents the measured CRF values of our cohort related to the different BMI values of the included patients with obesity; the *light blue halo* represents the 95% confidence interval. The *dashed lines* represent the predicted CRF of the different equations included in this study. Most equations lead to an overestimation of the CRF for patients belonging to obesity classes I and II and an underestimation for class III, especially with very high BMI.

The comparisons between the regression line obtained from the measured CRF cohort data (relative CRF ρ = -0.49, *P* < 0.001 and absolute CRF ρ = 0.81, *P* < 0.001) and the other 21 predicted CRF equations are shown in Figure 3A, B. Most of them overestimate measured CRF for BMI values close to 30 kg·m^−2^ and underestimate it for high BMI values.

**FIGURE 4:**
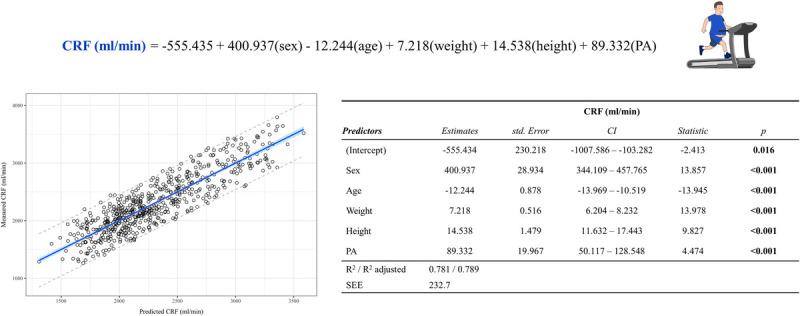
Novel CRF prediction equation for treadmill exercise in patients with obesity. The variables in the equation are defined as follows: sex (1: male, 0: female), age (yr), weight (kg), height (cm), and PA (0: inactive; 1: physically active, even if < 150 min·wk^−1^ of moderate-vigorous intensity physical activity). The graph represents the correspondence between measured and predicted CRF of the model. The *blue halo* shows the 95% confidence intervals, and the *dotted lines* represent 95% prediction intervals. The table shows the model for absolute CRF (mL·min^−1^). CI, confidence interval; PA, physical activity.

Finally, a multiple model for a treadmill exercise CRF prediction (expressed in mL·min^−1^) was generated from the selected cohort including age, sex, weight, height, and the physical activity level as determinants (Fig. 4). The linear model demonstrated a good fit (*R*^2^ = 0.78 and SEE = 232.7 mL·min^−1^). The performance of the new predictive equation was comparable to the overall estimate of the 10-fold and Monte Carlo cross-validations (average *R*^2^ = 0.785 and SEE = 233.750 mL·min^−1^ and *R*^2^ = 0.777 and SEE = 236.962 mL·min^−1^, respectively; Supplemental Table 7, Supplemental Digital Content 1, 10-fold internal cohort and Monte Carlo cross-validations, http://links.lww.com/MSS/D19).

## DISCUSSION

This study primarily aimed to examine the performance of predicted CRF equations in a large population of patients with obesity. Previously published predictive equations derived from general population cohorts, including those that are most used in clinical practice, were shown to incorrectly estimate CRF in patients with obesity. Therefore, our further objective was to develop and validate an equation that more accurately estimates the CRF in this specific population and to facilitate clinical CPET interpretation.

### CRF in patients with obesity

The global incidence of obesity has almost tripled worldwide since 1975 (29), and the WHO has been alarming for years about the growing state of the obesity pandemic in Europe and the United States (6,30). Therefore, in the Western world, a large portion of the population is affected by obesity, and consequently, it is necessary that health systems adapt to this trend when possible, considering the presence of this condition in every form of clinical assistance, including CRF estimation and measurement. Indeed, CRF is a strong predictor of cardiovascular and all-cause mortality in both healthy subjects and patients with chronic diseases (31). CRF could be particularly helpful in patients with obesity as, despite the obesity paradox (32), their cardiovascular risk is generally higher compared with normal-weight subjects, and it could also be used in the preoperative risk assessment of patients undergoing different invasive treatments, including bariatric surgery (33,34).

A 2016 scientific statement from the American Heart Association affirmed that clinicians should routinely include CRF evaluations in risk stratification, using also predictions with nonexercise equations when measured CRF is not available and CPET not feasible (2). Furthermore, various recommendations state that the ideal reference values for CPET should originate from a study group comparable to the one under examination, considering different essential parameters of that specific population (35,36). However, most used CRF reference values in clinical practice have emerged from healthy general population cohorts including, as majority, normal-weight individuals. Some authors have proposed CRF prediction equations specifically for patients with obesity, but they were generated from small cohorts and/or not adopted in current clinical practice (10,11,37).

### Predicted CRF versus measured CRF

The present study compared predicted CRF as determined by multiple nonexercise prediction equations to measured CRF within a large cohort of patients with obesity. This study has intentionally included only equations having variables associated with the weight of the subjects to minimize the difference between predicted and measured CRF. The Debeaumont et al. (10) equation, although specifically developed for patients with obesity, was excluded as it was only valid for women with metabolic syndrome, not considering the weight of the participants. The Barbosa et al. (11) equation was not included as it did not meet the inclusion criteria regarding available variables.

The estimated CRF values from the included equations were all significantly correlated with the measured CRF, although differences in the *R*^2^ and SEE values were observed. The SEE of the included equations for the overall sample was between 2.84 and 3.26 mL·kg^−1^·min^−1^, values roughly approximate to 1 MET. Different studies demonstrated a clear relationship between risk reduction and METs increase in nonexercise-predicted CRF for all-cause and cardiovascular mortality (2); thus, most of the included equations can wrongly influence the risk classification of patients with obesity. Furthermore, all equations showed a lower accuracy in estimating CRF in this cohort compared with the original publications with even lower mean *R*^2^ for the sex, BMI, age, and CRF-specific subgroups. In contrast to what emerged in the general population (5,38), equations including the physical activity level did not show better correlations between predicted and measured CRF. This lack of difference may be explained because most of the subjects in our sample can be considered totally sedentary. Even those who engage in regular physical activity achieve an average level that is insufficient according to international guidelines, considering both aerobic and strength training/activities (39,40).

### The relationship between BMI and CRF

The inverse relationship between BMI and relative CRF is known and well established, and our study confirms this even for very high BMI values. In fact, the majority of patients with obesity within the lowest CRF tertile belong to class III (79%). Interestingly, most of these equations, as evidenced by the Bland–Altman plots, did not show a cloud-like pattern toward a positive distribution between predicted and measured CRF. Not being conceived and generated for patients with obesity, the influence of high BMI values ​​seems to be excessive in most of these equations, thus leading to an underestimation of the real measured CRF, and even to negative relative values for patients with extremely high BMI (>60 kg·m^−2^; see Fig. 3A). Furthermore, the predictive equations included showed lower accuracy for obesity class II/III when compared with obesity class I, despite the higher number of patients with moderate-severe obesity in our cohort. The reasons for this discrepancy may be different and not only related to gas exchange measurement but also to a higher energy cost of walking in patients affected by severe obesity (41). Indeed, with increasing BMI, some biomechanical factors not present in the CRF equations such as reduced stability, a wider supporting base, and excessive lateral leg swing need to be considered. In addition, most patients belonging to obesity class III have relevant weight-induced comorbidities, including load arthropathies, which may significantly impact CRF (42).

Moreover, Figure 3B shows graphically that almost all equations were inaccurate even for absolute CRF, leading to an overestimation for mild to moderate obesity but to a progressive underestimation as BMI values raise. Interestingly, 14 out of 21 CRF prediction equations even showed a negative trend for absolute CRF, stressing even more how these equations were not appropriate for this specific population.

Two equations showed a good agreement with the measured CRF data for both relative and absolute CRF values and possessed unique features (16,28). The Wasserman et al. (16) equation is the only one of those included in this study to estimate absolute CRF values and to consider the difference between measured and ideal weight of the subjects, and hence, it seems suitable for patients with obesity. On the other hand, the allometric equation of Nevill et al. (28) is unique in proposing a nonlinear model. Although this equation is generated from the “Fitness Registry and the Importance of Exercise: A National Data Base” (FRIEND) registry, where the sample of subjects affected by obesity were only 25% (28), it demonstrated to well estimate CRF across all obesity classes. In fact, this equation has the highest average correct classification percentage among the obesity classes in our cohort (56%). The curvilinear nature of this equation and the use of a stature-to-body-mass ratio may be the reasons for this good predictive model with real-world clinical data.

### Novel model development and validation

The absence of specific equations designed for this population, particularly for patients with severe obesity, required the creation of a novel model to predict CRF that yields more accurate reference values for patients with obesity. Considering the influence that the weight of the subjects could exert on the determination of the relative CRF, particularly for this population with extreme BMI values, the equation was deliberately generated to estimate the absolute CRF. The choice of determinants was dictated by the literature on CRF and based on the other previous equations. The physical activity level was considered as a dichotomous variable (nonactive and active) for feasibility reasons in clinical practice. The *R*^2^ (0.78) could be considerate in high range with higher values than those reported for the included equations (0.43 to 0.74). The clinical application of this newly generated predictive equation may thus improve the clinical utility of predicted CRF in patients with obesity undergoing CPET during functional evaluation and subsequent risk prediction.

### Limitations and perspectives

This is the largest clinical trial analyzing the predicted CRF of different treadmill exercise equations specifically in patients with obesity, proposing and validating a specific new CRF equation for this population. However, different limitations need to be considered. (a) Primarily, even though the derivation sample is the largest used for the genesis of a model in patients with obesity, it is still limited compared with other previous derivation cohorts of normal-weight subjects. (b) Prediction equations considering body fat have not been included in this analysis as this variable is not systematically collected in our center. This resulted in the exclusion of some variants of equations still included in the study. Despite a previous work that showed that only small differences were observed between equations including BMI and those including percentage of body fat in healthy subjects (5), the prognostic importance of body fat determination to correctly interpret CRF has been highlighted in patients with different chronic conditions, including obesity (43,44). Indeed, because it is not easy to get valid data for patients with severe obesity, future studies focusing on patients with less severe obesity should incorporate these measures. (c) The ethnicity of patients in the present study was predominantly White, and some substantial differences emerged with equations developed in cohorts of different ethnicities where also obesity rates are lower compared with Western countries (18). Moreover, differences in lifestyle and baseline physical activity levels between populations could influence the generalizability of our findings. Thus, future research will need to describe the impact obesity may have on CRF in other ethnic cohorts. (d) More focus should be placed on genetics, as it is known that some genetic variants could influence CRF while others are associated with the predisposition for obesity. The influence of genetics was currently not considered in any predictive equation. (e) The use of different classifications for smoking history and physical activity level to convert into the various equations may have added a bias when compared with the data of the original publications. (f) Although we excluded patients with heart, vascular, and lung diseases, a significant proportion of patients were affected by frequent obesity-associated comorbidities whose effect on CRF is difficult to estimate. Other conditions such as metabolic, polycystic ovary, and obstructive sleep apnea syndrome were not investigated, and some of them appear to influence CRF (13). (g) Finally, this research project addressed only the treadmill as exercise modality. Cycle ergometer tests were deliberately excluded because in our center these tests are reserved for patients with mobility impairments. In addition, the cycle ergometer does not allow us to perform tests in patients with body mass above 160 kg, limiting the inclusion of patients with class III obesity. Future studies are needed to test the clinical validity and generate new equations for cycle ergometer testing in patients with obesity.

## CONCLUSIONS

The equations commonly used in clinical practice for CRF prediction in patients with obesity are not based on these specific populations. Indeed, most of these equations showed high variability and low accuracy, even further when subgroups are considered, especially those with very high BMI and/or low CRF. A new equation estimating CRF was generated for patients with obesity and validated for the clinical interpretation of CPET conducted during treadmill exercise.
